# Improving the Emotional Distress and the Experience of Hospitalization in Children and Adolescent Patients Through Animal Assisted Interventions: A Systematic Review

**DOI:** 10.3389/fpsyg.2022.840107

**Published:** 2022-03-04

**Authors:** Cinzia Correale, Marta Borgi, Barbara Collacchi, Chiara Falamesca, Simonetta Gentile, Federico Vigevano, Simona Cappelletti, Francesca Cirulli

**Affiliations:** ^1^Clinical Psychology Unit, Department of Neurosciences, Bambino Gesù Children’s Hospital, IRCCS, Rome, Italy; ^2^Center for Behavioral Sciences and Mental Health, Istituto Superiore di Sanità, Rome, Italy; ^3^Department of Humanities, LUMSA University, Rome, Italy; ^4^Department of Neurosciences, Bambino Gesù Children’s Hospital, IRCCS, Rome, Italy

**Keywords:** animal-assisted interventions, hospitalization, pediatric patients, stress, pain, anxiety, children, adolescents

## Abstract

**Introduction:**

Animal Assisted Interventions (AAIs) are increasingly common in pediatric care settings as a means to promote the physical, mental, and emotional well-being of hospitalized children and adolescents.

**Objectives:**

The aim of this work was to review published studies implementing AAIs in hospital settings and to assess the effects of AAIs on the biobehavioral response to stress and pain, social behavior, quality of life and level of satisfaction with hospitalization in children and adolescents. Stress and burden, quality of life, mood and level of satisfaction with hospitalization in parents/caregivers as well as stress and burden, perception of the work environment and job satisfaction in hospital staff were also reviewed.

**Methods:**

All published studies reporting quantitative assessments were systematically searched using PubMed, Scopus, ProQuest and Web of Science databases in accordance with PRISMA guidelines. The aim was to identify studies examining the effects of AAIs on behavioral, psychological and physiological responses to stress in children and adolescents (0–18 years) formally admitted to a hospital for a stay, as well as in those undergoing a visit for treatments or medical examinations.

**Results:**

Of the 350 studies screened, 21 were eligible for inclusion. Most of them focused on stress, pain, and anxiety reduction in pediatric patients, and used both physiological parameters and behavioral and psychological observations/scales. All studies employed dogs. Results show the potential of AAIs to reduce anxiety and behavioral distress in pediatric patients while acting on physiological measures associated with arousal.

**Conclusion:**

Although further, more rigorous studies are still needed, the findings of this review may have implications for clinical practices suggesting appropriate planning of AAIs by pediatric healthcare professionals.

**Systematic Review Registration:**

[https://www.crd.york.ac.uk/prospero/display_record.php?RecordID=178993], identifier [CRD42020178993].

## Introduction

Hospitalization is a stressful event for children and their families (Coyne, 2006; Francischinelli et al., 2012): as previously reported (Fernandes and Arriaga, 2010), separation from parents and friends, being in an unfamiliar environment and receiving procedures and treatments is a major concern for mental health in this patient population. Lack of control over the environment can be traumatic, as demonstrated by increased anxiety, aggression, anger, and similar emotional expressions in hospitalized children (Coyne, 2006; William Li et al., 2007; Francischinelli et al., 2012; Li et al., 2016). This condition could delay treatment, or lead to a longer time to recover, also reducing patient and family satisfaction. Traditionally, pharmacological therapies have been prescribed to manage anxiety and stress in this condition, but this is often associated with high costs and harmful side effects, including constipation and nausea (Jiang et al., 2004; Ravindran and da Silva, 2013; Taniguchi et al., 2015). Therefore, the advent of non-pharmacologic approaches based on emotional regulation and humanization of care through complementary therapies has drawn the attention of the medical community (Connor and Miller, 2000; Ravindran and da Silva, 2013; Taniguchi et al., 2015; Gilmer et al., 2016). Among complementary interventions, Animal-Assisted Interventions (AAIs) appear to represent a highly suitable approach that could be implemented in children’s education and care (Cirulli et al., 2011; Davis et al., 2015; Correale et al., 2017). The term AAIs refers to goal-oriented and structured interventions that incorporate domesticated animals in health, education, and recreational activities and are designed to promote improvement in human physical, social, emotional, and/or cognitive functioning (Kruger and Serpell, 2010). AAIs can be defined, as a function of their main goal, as Animal-Assisted Therapy (AAT), Animal-Assisted Education (AAE), and Animal-Assisted Activities (AAA). In Italy, AAI are conducted according to specific Guidelines from the Ministry of Health which regulate the involvement of health professionals into interdisciplinary teams, the planning and monitoring of the interventions, and the training (Cirulli et al., 2011; Italian National Guidelines for Animal Assisted Interventions, 2015).

Children’s hospitals are an elective setting where AAIs could be successfully employed, as indicated by an increasing number of studies (Kaminski et al., 2002; Barker et al., 2015; Abrahamson et al., 2016; Uglow, 2019). Although AAI effectiveness has been examined by previous systematic reviews and meta-analyses (Urbanski and Lazenby, 2012; Chur-Hansen et al., 2014; Gilmer et al., 2016; Lundqvist et al., 2017; Waite et al., 2018; Tripodi et al., 2019; Feng et al., 2021), the focus of these studies was not always on the hospital setting or, alternatively, it was limited to a specific medical condition (such as pediatric oncology). Furthermore, they were limited to randomized controlled studies. Hence, we here provide an update on AAI’s effectiveness in pediatric wards considering a variety of outcomes, including bio-behavioral responses to stress and pain (e.g., anxiety, cortisol levels, perceived stress, and pain), mood (e.g., depression), social behavior, quality of life and level of satisfaction with hospitalization. In order to provide a broader overview of children hospital application of AAIs, we included non-controlled studies, but only if they included quantitative scales for data collection. Furthermore, we sought to assess whether these interventions can also impact stress and burden, quality of life, mood and level of satisfaction in parents and/or caregivers, as well as ameliorating the work environment and promote job satisfaction, as perceived by the hospital staff. These indicators could be important to further promote AAIs in hospital settings.

## Materials and Methods

### Review Protocol

The systematic search was conducted in accordance with the Preferred Reporting Items for Systematic Reviews and Meta-Analyses (PRISMA) guidelines (Liberati et al., 2009; Moher et al., 2009). The protocol (based on PRISMA-P checklist; Moher et al., 2015; Shamseer et al., 2015) was registered in PROSPERO registry on Jul 05, 2020 (registration number: CRD42020178993).

### Literature Search and Study Selection

Relevant literature was searched in: Elsevier’s Scopus, Pubmed, Web of Science (Core Collection), and ProQuest (Biological Science Collectionı, British Nursing Databaseı, Health & Medical Collection, and Psychology Database). Manual search was then performed in order to supplement primary database searches. Searches were conducted on 14th April 2020. All studies reporting quantitative assessment and published in peer review journals were included.

The search was aimed at identifying relevant studies examining the effects of AAIs on the following outcomes: (1) biobehavioral response to stress and pain (e.g., anxiety, cortisol levels, perceived stress, and pain), mood (e.g., depression), social behavior, quality of life and level of satisfaction with hospitalization in children and adolescents (age range: 0–18 years); (2) stress and burden, quality of life, mood and level of satisfaction with hospitalization in parents/caregivers; (3) stress and burden, perception of the work environment and job satisfaction in hospital staff. Studies were included if they assessed the effectiveness of AAIs in improving the experience of hospitalization both in children and adolescents formally admitted to a hospital for a stay (hereinafter referred to as “hospitalized” or “inpatients”), as well as in those who attend a hospital for a visit (i.e., for treatments or medical examinations) (hereinafter referred to as “outpatients”). For this study, AAIs were defined as scheduled visits of an animal accompanied by its handler to the hospital and involving domestic animals (interventions involving residential animals as well as wild animals, such as dolphins, were excluded). The complete search strategy used is presented in the Supplementary Material (example of Scopus database, Supplementary Material).

Titles/Abstracts and full text of studies retrieved were screened independently by two authors (BC, CC). A rater agreement of 88.9 and 91.9% was reached between the two reviewers in the Title/Abstract and Full text screening phases respectively. Any disagreement was solved through discussion with an additional investigator (MB). The prioritization of exclusion criteria was: (i) language different from English; (ii) non-original research (e.g., reviews, editorials, commentaries) (iii) interventions other than AAIs in hospital settings; (iv) AAIs involving non-domestic animals; (v) subjects receiving the interventions different from children and adolescent (range: 0–18 years of age); (vi) not relevant outcomes; (vii) qualitative assessment, descriptive statistics; (viii) no full-text available.

The full text of the potentially eligible studies was requested from corresponding authors by email. If there was no response to our initial email, after a minimum of five business days, we sent a second reminder email to the corresponding author.

### Data Extraction

The full-text articles of the studies eligible for qualitative data extraction were independently assessed by two reviewers (BC, CC) with discrepancies that could not be resolved by discussion being solved by consulting an additional investigator (MB).

The data extracted included the following categories: (i) bibliographic details (i.e., 1st author, country, year of publication, journal); (ii) subject characteristics (i.e., age, sex, diagnosis, concomitant medications/behavioral interventions); (iii) care setting (i.e., inpatient/outpatient units, duration of hospitalization, reasons for being hospitalized or type of visit); (iii) AAI characteristics (i.e., animal species involved and number, intervention duration/frequency, activity performed, team involved, other subjects involved such as parents or hospital staff); (iv) study design characteristics (i.e., experimental groups receiving AAI and age-matched controls receiving another behavioral intervention or treatment as usual, sample sizes, follow-up, randomization). Outcome measures extracted were: (1) changes in biobehavioral response to stress and pain, mood, social behavior, quality of life, and level of satisfaction with hospitalization in children and adolescents; (2) changes in stress and burden, quality of life, mood, and level of satisfaction with hospitalization in parents/caregivers; (3) changes in stress and burden, perception of the work environment and job satisfaction in hospital staff. In particular, we retrieved data on the direction of the variation, i.e., statistically significant improvement, worsening, no change. Other measures extracted were: (i) other outcomes; (ii) level of satisfaction with the intervention in children/adolescents, and caregivers/parents; (iii) measurements assessing children-animal interaction/relationship and children’s attitudes toward animals.

### Assessment of the Risk of Bias

The revised Cochrane risk-of-bias tool for randomized trials (RoB2, Sterne et al., 2019) was used to assess the risk of bias in randomized studies. The methods of randomization, deviations from intended interventions (effect of adhering to intervention), missing outcome data, measurement of the outcome, selection of reported results in the included studies were evaluated. Each domain was judged as “low,” “some concerns,” or “high” risk based on responses to signaling questions, resulting in an overall bias judgment being assessed.

The ROBINS-I tool (Risk Of Bias In Non-randomized Studies - of Interventions; Sterne et al., 2016) was used in the case of non-randomized studies of interventions. The tool comprises seven domains (confounding, selection of participants into the study, classification of interventions, deviations from intended interventions, missing data, measurement of outcomes, selection of the reported results) and an overall judgment of risk of bias. Risk of bias for each domain and the overall judgment can be expressed as “low,” “moderate,” “serious,” critical,” or “no information.”

Two reviewers (BC, CC) independently assessed risk of bias in selected studies; any disagreement was resolved through discussion, and the involvement of a further reviewer (MB), if required.

## Results

### Study Selection

**FIGURE 1:**
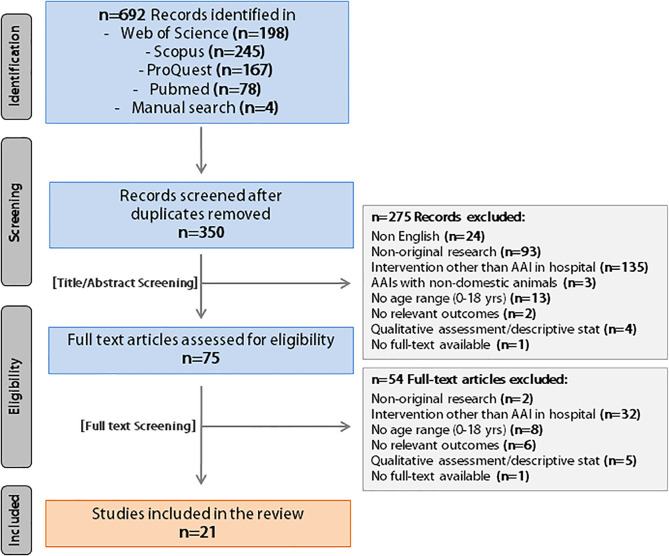
PRISMA flow diagram of the literature search (identification) and selection process (screening, eligibility, inclusion).

The comprehensive search strategy resulted in 692 bibliographic records. The study selection process is summarized in Figure 1 by using the PRISMA flow diagram. After duplicates were removed, 350 studies were left. The 1st selection phase (i.e., titles and abstracts screening) resulted in 75 studies; the 2nd selection phase (i.e., full-text articles screening) resulted in 21 studies eligible for inclusion in the systematic review.

### Study Characteristics

Of the 21 studies included in the analysis, 12 were conducted in the United States of America (57%), three in Italy (14%), two in Canada (10%), and four in other countries (19%). Patients were children aged between three months and 18 years, with a balance between males (*n* = 471) and females (*n* = 458) (Table 1). Almost half of the sample (48%) were outpatients while 52% were inpatients. Hospitalization reasons for inpatients were medical or surgical management (64%) or surgical procedures (36%). Visit reasons for outpatients were routine examination (30%), oncological treatment (20%), dental visit (20%), and others (30%). Types of medical conditions ranged from chronic health conditions (i.e., psoriatic arthritis, tuberous sclerosis, Prader-Willi Syndrome, cystic fibrosis, diabetes, neurological disorders) to acute illness (i.e., fever, otitis media, tick bite, trauma), oncological disorders, dental issues, neurodevelopmental disorders or gastrointestinal diseases. Two studies included healthy patients. The majority (72%) of the studies included a control group, but only 38% of them has a random assignment. Sample size ranged from 15 to 60 subjects for AAI group and from 15 to 81 subjects for control groups. The more frequent outcome assessed was the child’s stress (*n* = 14 studies), followed by anxiety (*n* = 7) and pain (*n* = 8). Other outcomes (i.e., mood and QoL) were less frequently studied. Only three of 21 studies considered also parents’ outcomes (anxiety, stress, and mood). None of the studies selected assessed stress and burden, perception of the work environment, or job satisfaction in hospital staff.

**TABLE 1:** Characteristics of the included studies.

Bibliographic details		Care setting			Subjects			Study design		
Authors, year	Country	Inpatient/ Outpatient	Hospitalization: reason	Visit: reason	Age range (mean ± SD)	Sex (M/F)	Medical condition	Type	Sample size	Population: Outcomes
Ávila-Álvarez et al., 2020	Spain	Outpatients	–	Therapeutic care and Rehabilitation	2.5–5.5 years (3.9 ± 12.6)	13/6	Autism spectrum disorder	Uncontrolled	**AAI:** 19	**Child:** Communication and Interaction skills
Antonelli et al., 2019	Italy	Outpatients	–	Short-term treatment or observation	3–16 years (EG*: 8.2 ± 3.3; **Ctrl:** 8.3 ± 4.1)	EG*: 31/26; **Ctrl:** 23/25	Respiratory, Gastrointestinal Urinary tract and Neurological diseases; Traumatic pathology	Controlled Randomized	**AAI:** 24; **Ctrl:** 81**	**Child:** Pain
Hinic et al., 2019	United States	Inpatients	Medical or surgical management	–	6–17 years (**AAI:** 11.00 ± 3.46; **Ctrl:** 10.05 ± 3.17)	**AAI:** 21/29; **Ctrl:** 19/24	Acute Infection, Chronic Illness, Neurologic and Gastrointestinal conditions	Controlled	**AAI:** 50; **Ctrl:** 43	**Child:** Anxiety
Lindström Nilsson et al., 2020	Sweden	Inpatients	Surgical procedures (neurology, orthopedic, gastro, and urology surgery)	–	3–18 years (11.5 ± 3.97)	24/26	Brain cancer; Brain damage; Neurologic condition; Trauma	Uncontrolled	**AAI:** 50	**Child:** Wellbeing; Experience of hospital stay
Perez et al., 2019	Canada	Outpatients	–	MRI (magnetic resonance imaging)	5.1–16.5 years (median: 8 years)	11/10	Autism, hearing loss, developmental delay, tuberous sclerosis, psoriatic arthritis, urinary incontinence and spastic diplegia.	Uncontrolled^§^	**AAI:** 21	**Child:** Anxiety
Nammalwar and Rangeeth, 2018	India	Outpatients	–	Dental visit	4–11 years	9/11	Healthy	Uncontrolled	**AAI:** 20	**Child:** Anxiety
McCullough et al., 2018	United States	Outpatients	–	Oncological treatment	3–17 years (**AAI:** 8.9 ± 4.5; **Ctrl:** 8.1 ± 4.6)	**AAI:** 31/29; **Ctrl:** 26/20	Leukemia, Lymphoma, Sarcoma, Other	Controlled Randomized	**AAI:** 60; **Ctrl:** 46	**Child:** Stress#, Anxiety, QoL **Parents:** Stress
Silva and Osório, 2018	Brazil	Outpatients	–	Oncological treatment	6–12 years (8.68 ± 1.98)	10/14	Leukemia and solid tumors	Uncontrolled	**AAI:** 24	**Child:** Stress, Pain, Depression, Mood, QoL **Parents:** Mood, Anxiety
Branson et al., 2017	United States	Inpatients	Surgery	–	7–17 years (**AAI:** 13.43 ± 0.59; **Ctrl:** 12.83 ± 0.58)	**AAI:** 13/11; **Ctrl:** 11/13	Trauma, gastrointestinal, and musculoskeletal disorders	Controlled Randomized	**AAI:** 24; **Ctrl:** 24	**Child:** Stress#; Anxiety
Chubak et al., 2017	United States	Inpatients	Medical or surgical management	–	7–18 years (12.9 ± 3.6)	10/9	Leukemia, Lymphoma, Sarcoma, Brain cancer	Uncontrolled	**AAI:** 19	**Child:** Stress, Pain
Barker et al., 2015	United States	Inpatients	Medical or surgical management	–	8–17 years (11.83)	19/21	31 different conditions. Most frequently reoccurring (*n* = 3) appendicitis and abdominal pain	Controlled Randomized	**AAI:** 20; **Ctrl:** 20	**Child:** Pain, Anxiety
Calcaterra et al., 2015	Italy	Inpatients	Surgical procedures (orchidopexy, inguinal or umbilical hernia repair, circumcision, varicocele treatment)	–	4–16 years (**AAI:** 8.59 ± 3.70; **Ctrl:** 7.36 ± 2.48)	**AAI:** 15/5; **Ctrl:** 17/3	Healthy	Controlled Randomized	**AAI:** 20; **Ctrl:** 20	**Child:** Stress#, Pain
Vagnoli et al., 2015	Italy	Outpatients	–	Blood testing (routine exams)	4–11 years (**AAI:** 7.1 ± 1.8; **Ctrl:** 7.4 ± 2.5)	**AAI:** 12/13; **Ctrl:** 12/13	Healthy	Controlled Randomized	**AAI:** 25; **Ctrl:** 25	**Child:** Stress, Pain **Parents:** Anxiety
Tsai et al., 2010	United States	Inpatients	Medical or surgical management	–	7–17 years (10.97 ± 3.01)	7/8	Acute or chronic conditions	Controlled (within-subject)	**AAI/Ctrl:** 15	**Child:** Stress#, Anxiety
Braun et al., 2009	United States	Inpatients	Medical or surgical management	–	3–17 years (**AAI:** 13.00 ± 4.01; **Ctrl:** 11.69 ± 4.61)	**AAI:** 7/11; **Ctrl:** 22/17	Acute and Chronic illnesses	Controlled	**AAI:** 18; **Ctrl:** 39	**Child:** Stress#, Pain
Sobo et al., 2006	United States	Inpatients	Surgery	–	5–18 years	9/16	n.a.	Uncontrolled	**AAI:** 25	**Child:** Stress, Pain
Kaminski et al., 2002	United States	Inpatients	Medical or surgical management	–	≥ 5 years (9.86 ± 2.80)	39/31	Hematological and oncological disorders, cystic fibrosis, diabetes, and transplants	Controlled	**AAI:** 30; **Ctrl:** 40	**Child:** Stress#, Mood
Wu et al., 2002	Canada	Inpatients	Medical or surgical management	–	3 months-16 years (median: 7 years)	20/10	Cardiac and Non-cardiac conditions	Uncontrolled	**AAI:** 30	**Child:** Stress#
Havener et al., 2001	United States	Outpatients	–	Dental procedure	7-11 years (**AAI:** 8.4 ± 1.23; **Ctrl:** 8.85 ± 1.04)	17/23	Dental conditions requiring fillings, extractions, crown placements, sealants, cleanings.	Controlled Randomized	**AAI:** 20**; Ctrl**: 20	**Child:** Stress
Hansen et al., 1999	United States	Outpatients	–	Routine physical examination	2–6 years (**AAI:** 4.1 ± 0.9; **Ctrl:** 3.5 ± 1.5)	**AAI:** 5/10; **Ctrl:** 9/10	Otitis media, Fever. Headache, Asthma, Prader-Willi Syndrome, Tick bite, Follow-up from a fall	Controlled Randomized	**AAI:** 15; **Ctlr:** 19	**Child:** Stress
Nagengast et al., 1997	United States	Outpatients	–	Routine physical examination	3–6 years (4.7 ± 1.01)	9/14	Healthy	Controlled (within-subject)	**AAI/Ctrl**: 23	**Child:** Stress

*EG: experimental Group (includes three different interventions: AAI, clowns, and music in hospital).

**In this study (Antonelli et al., 2019) the Ctrl group was further split in three groups based on the activity performed: clowns (n = 18), Musicians (n = 15), No intervention (n = 48).

*^§^ Controlled only for the following outcomes: for the Completion of examinations, exam quality, and average exam time.*

*# Only physiological measures.*

*AAI, Animal-Assisted Intervention; Ctrl, Control Intervention; n.a., not available; QoL, Quality of Life.*

All selected studies used only dog-mediated interventions; we found no other species involved in AAIs pediatric hospital programs. For what concerns the interventions (Table 2), programs included: (i) a single dog visit (*n* = 15), (ii) regular (weekly) dog visits (*n* = 5), or (iii) dog visits at any time during the patient’s hospitalization (*n* = 1). Sixteen of 21 studies specified the duration of the session, which results highly variable (range 6–60 min; *M* = 16.44; *SD* = 8.29). The number of sessions in regular interventions varied also for number of encounters (range 3–24 sessions, *M* = 14.4; *SD* = 7.55). The smallest AAI team included a dog with its handler. Fifteen of the selected studies mentioned the breed of the dog employed, mainly represented by Retrievers (Golden or Labrador) or Retrievers mixes (*n* = 40). Other breeds employed were Shelties (*n* = 2), Shi Tzu (*n* = 2), Small Mongrels (*n* = 2), and others (*n* = 11). Only 11 studies mentioned also the handler qualification, which results to be mostly an AAI’s trained or expert (64%). Except for seven studies, activities with the dog were mainly organized in individual sessions (*n* = 14) and usually involved free/naturalistic interaction (38%) or petting/caring for the dog (29%). Control group interventions were highly variable (see Table 2 for more details on the animal-assisted and control interventions).

**TABLE 2:** Intervention characteristics of the included studies.

Authors, year	AAI Intervention	AAI team	AAI Activities	Ctrl Intervention
Ávila-Álvarez et al., 2020	Regular dog visits: 9 sessions (20 min/once a week)	5 dogs (3 Labrador Retrievers, 1 Galician Shepard Dog, 1 Spanish Water Dog); Handler: occupational therapist with AAI training	Individual session: activities focusing on the knowledge of the dog, interaction with the dog, the care of the animal, and playful occupations with a primary focus on social interactions	–
Antonelli et al., 2019	Dog visit: single session (n.a.)	n.a.	Free/naturalistic interaction with the dog: professionals determined the type and timing of the activities based on their experience	Standard care only
Hinic et al., 2019	Dog visit: single session (8-10 min)	2 dogs (1 Labrador and 1 Golden retriever); Handler with AAI training	Individual session: Free/naturalistic interaction with the dog followed by a brief coping skills education designed by the child-life specialist	Completion of a simple age-appropriate jigsaw puzzle followed by a brief coping skills education designed by the child-life specialist
Lindström Nilsson et al., 2020	Dog visit: single session (n.a.)	1 dog (Labradoodle); Handler: qualified dog trainer	Individual session: The interaction started with a calm period and after that an active period with dog tricks guided by the handler. A period of relaxation concluded the therapy. Finally, each child received a stuffed toy resembling the dog	–
Perez et al., 2019	Visit (MRI) with the dog: single session (20-60 min)	1 dog (Labrador retriever); Handler	Individual session in the waiting room (before the scan): Sitting near the dog, petting it, and engaging in low-level play under the supervision of the professional trainer	–
Nammalwar and Rangeeth, 2018	Visit (dental procedure) with the dog: single session (15 min)	n.a.	Individual session: dog in the waiting room and during dental treatment. Dog allowed sitting on patients	–
McCullough et al., 2018	Regular dog visits: 4 months (10–20 min/once a week)	26 dog (Labradors and Labrador mixes); Handlers with AAI training	Group session: Free/naturalistic interaction with the dog	Standard care only#
Silva and Osório, 2018	Regular dog visits: 3 sessions (30-min/once a week)	2 dogs (1 Labrador retriever, 1 Golden retriever); Handler: physical therapist with AAI training	Open group (max 7 participants): (1) Sensory stimulation: sensorial and upper limb stimulation (brush, pet, and play fetch with the dog); (2) Gait training: training on activities of daily living (give water and food to the food) and gait (walking with the dog); (3) Socialization and Recreation: dog show, playing with the dog’s supplies, dog drawing; agility courses, dog clothes, stories about the dog	–
Branson et al., 2017	Regular dog visits: 10 months (10 min/twice per month)	9 dogs (1 Standard poodle, 1 English mastiff, 1 Yorkshire Terrier, 1 Shih tzu, 1 Schnauzer, 1 Pug, 1 Golden retriever, 2 Shelties); Handlers	Free/naturalistic interaction with the dog	Free/naturalistic interaction with the plush stuffed dog
Chubak et al., 2017	Dog visit: single session (20 min)	1 dog; Handler with AAI training	Individual session: the handler sat in a chair next to the bed, provided hand sanitizer to the patient, and invited him/her to pet the dog. The handler talked with the patient and family and often invited the dog to show the patient a trick. At the end of the visit, the handler provided the patient with her dog’s “business card,” which included a photo, to provide children with a keepsake	–
Barker et al., 2015	Dog visit: single session (10 min)	7 dogs; Handlers with prior experience visiting pediatrics	Free/naturalistic interaction with the dog	Completion of an age-appropriate jigsaw puzzle
Calcaterra et al., 2015	Dog present during post-operative awakening (2 hours after surgery): single session (20 min)	1 dog (Golden retriever); Handler	Individual session: dog present during post-operative awakening, at re-admission to the Unit	Standard care during post-operative awakening
Vagnoli et al., 2015	Visit (blood testing) with the dog: single session (15 min)	4 dogs (1 Labrador, 1 mixed-breed, and 2 small mongrels); Handler: AAI expert	Individual session: the dog accompanied the child (and his/her parent) in the procedure room during the venipuncture	Visit without a dog
Tsai et al., 2010	Dog visit: single session (6-10 min)	1 dog; Handler with AAI training	Individual session: the child was allowed to pet, touch, and brush the dog	Completion of an age-appropriate puzzle
Braun et al., 2009	Dog visit: single session (15-20 min)	1 dog (Springle Spaniel); Handler with AAI training	Individual session: Free/naturalistic interaction with the dog	Sitting quietly
Sobo et al., 2006	Dog visit: anytime during hospitalization, the patient generally determines the length of the intervention	1 dog (West Highland White Terrier); Handler: clinical nurse specialist	Individual session: the patient decided the level of interaction (passive: dog sitting or sleeping with the child; low: dog doing an occasional pet trick; high: active, playful roughhousing and going for walks with the child)	–
Kaminski et al., 2002	Dog visit: single session (n.a.)	n.a.	Group session: Free/naturalistic interaction with the dog	Group activities (e.g., working on structured crafts or other projects, and playing games or cards) or individual activities (e.g., playing video games)
Wu et al., 2002	Regular dog visits: 6 months (10-20 min/once a week)	3 dogs (1 Golden Retriever, 1 Shi Tzu; 1 mixed breed); Handlers with AAI training	Individual session: Free/naturalistic interaction with the dog: during the visit, both the patient and parent were free to interact creatively with the dogs in any manner they wish under the supervision of trained volunteers	-
Havener et al., 2001	Visit (dental procedure) with the dog: single session	1 dog (Golden retriever)	Individual session: the child was encouraged to pet, touch, and talk to the dog as desired during the dental procedure	Dental procedure without the dog
Hansen et al., 1999	Visit (physical examination) with the dog: single session (2–15 min)	1 dog (Golden retriever)	Free/naturalistic interaction with the dog	Physical examination without the dog
Nagengast et al., 1997	Visit (physical examination) with the dog: single session (10 min)	1 dog (Beagle)	Individual session: the dog was brought into the room and positioned on the examination table next to the child	Physical examination without the dog

*^#^Participants in the control group were not prohibited from having interactions with the AAI team who happened to be onsite, such as a brief interaction in the waiting room or hallway.*

*AAI, Animal-Assisted Intervention; Ctrl, Control Intervention; n.a., not available.*

### Effect of Animal Assisted Interventions on Stress in Hospitalized Children and Adolescents (Subjective Outcomes)

Seven of the selected studies examined the effects of AAI on behavioral stress responses. As listed in Table 3A all the controlled trials measured the outcome with the Observation Scale of Behavioral Distress (OSBD). In this scale, independent raters observe the patient for behavioral signs of distress such as crying, screaming. Both Nagengast et al. (1997) and Hansen et al. (1999) found a significant improvement in stress levels during a physical examination when a dog was present, compared to not present. Vagnoli et al. (2015) found an improvement in perceived stress levels before and during the interaction with the dog, compared to the control group, while finding no modification after the interaction was over. Havener et al. (2001) examined the effect of the AAI during a dental procedure and found no significant modification on the outcome during the interaction. In all uncontrolled trials, as shown in Table 3B, a significant reduction in perceived stress levels was found, measured using different questionnaires/scales before and after the intervention.

**TABLE 3A:** Effect of AAI on behavioural response to stress (controlled studies).

Study	Assessments/ Analysis	Instruments/tool	Results
Vagnoli et al., 2015 ^#^	AAI vs. Ctrl (Before-During-After intervention)	OSBD	Before: ↑ During: ↑ After: =
Havener et al., 2001 ^#^	AAI vs. Ctrl (During intervention)	OSBD	=
Hansen et al., 1999 ^#^	AAI vs. Ctrl (Baseline-During intervention)	OSBD	↑
Nagengast et al., 1997	AAI vs. Ctrl (During intervention)	OSBD	↑


**TABLE 3B:** Effect of AAI on behavioral response to stress (uncontrolled studies).

Silva and Osório, 2018	Pre–Post Intervention	CSSI	↑
Chubak et al., 2017	Pre–Post Intervention	PedsQL	↑
Sobo et al., 2006	Pre–Post Intervention	VAS	↑

*^#^Randomized controlled.*

*AAI, Animal-Assisted Intervention; Ctrl, Control Intervention; OSBD, Observation Scale of Behavioral Stress (Jay and Elliott, 1986); CSSI, Child Stress Symptoms Inventory (Lipp and Lucarelli, 1998); PedsQL, Present Functioning Scales – Emotional Stress summary score (Sherman et al., 2006); VAS, Visual Analog Scale (Foster and Varni, 2002).*

*↑, Statistically significant improvement; =, not significant changes.*

### Effect of Animal Assisted Interventions on Pain in Hospitalized Children and Adolescents (Subjective Outcomes)

Many studies have described the effects of AAI on perceived pain using the Wong-Baker Scale (Faces Scale) (Tables 4A,B). Although all the uncontrolled trials revealed a significant pre-post intervention effect on the perceived pain (Table 4B), when the study design involved a control group, only two of the five studies showed a significant improvement (Braun et al., 2009; Calcaterra et al., 2015; see Table 4A).

**TABLE 4A:** Effect of AAI on pain (controlled studies).

Study	Assessments/Analysis	Instruments/tool	Results
Antonelli et al., 2019 ^#^	AAI vs. Ctrl1, Ctrl2, Ctrl3 (only Post Intervention)	WBS	=
Calcaterra et al., 2015 ^#^	Group × Time. Pre (T1, post-operatively baseline) and Post (T2, after 20 min) Intervention	WBS	↑
Barker et al., 2015 ^#^	AAI vs. Ctrl: Pre–Post Intervention	NRS-11	=
Vagnoli et al., 2015 ^#^	AAI vs. Ctrl (After intervention)	WBS/VAS	=
Braun et al., 2009	Group × Time (Pre–Post Intervention)	WBS	↑

**TABLE 4B:** Effect of AAI on pain (uncontrolled studies).

Silva and Osório, 2018	Pre–Post Intervention	WBS	↑
Chubak et al., 2017	Pre–Post Intervention	PedsQL	↑
Sobo et al., 2006	Pre–Post Intervention	VAS	↑

*^#^Randomized controlled.*

*AAI, Animal-Assisted Intervention; Ctrl, Control Intervention; WBS, Wong- Baker Scale (Faces Scale) (Wong and Baker, 1988); NRS-11, Numerical Rating Scale – 11-point, single item for pain (von Baeyer et al., 2009); VAS, Visual Analog Scale (Foster and Varni, 2002); PedsQL, Present Functioning Scales – Pain item (Sherman et al., 2006).*

*↑, Statistically significant improvement; =, not significant changes.*

### Effect of Animal Assisted Interventions on Stress and Pain in Hospitalized Children and Adolescents (Physiological Outcomes)

Nearly half of the studies selected in this review focused on AAI effects on stress and pain levels by considering physiological outcomes. We summarized all findings on controlled and uncontrolled studies in Tables 5A,B. Eight studies, six controlled and two uncontrolled, measured the heart rate, a parameter that is affected by physiological and pharmacological stimuli (Nagengast et al., 1997; Hansen et al., 1999; Kaminski et al., 2002; Wu et al., 2002; Tsai et al., 2010; Calcaterra et al., 2015; McCullough et al., 2018; Silva and Osório, 2018). Results are mixed: three controlled and two uncontrolled studies found no significant changes, while Kaminski et al. (2002) and Calcaterra et al. (2015) found a statistically significant increase in heart rate activity. According to Calcaterra et al. (2015), the autonomic cardiovascular changes in heart rate could be considered as adaptative responses, while Kaminski et al. (2002) assumed that the increasing levels of heart rate activity might reflect an excitatory response of a group of patients when exposed to the dog. Only one study (Nagengast et al., 1997) found a significant decrease in heart rate when the companion animal was present during medical examination, meaning that it can also work inhibiting the sympathetic nervous system activity.

**TABLE 5A:** Effect of AAI on stress and pain (physiological outcomes) (controlled studies).

Study	Assessments/Analysis	Measures	Results
McCullough et al., 2018 ^#^	Group × Time (Pre–Post intervention)	Blood pressure	=
		Heart rate	=
Branson et al., 2017 ^#^	Group × Time (Pre-Post intervention)	Salivary cortisol	=
		C-reactive protein	=
Calcaterra et al., 2015 ^#^	AAI vs. Ctrl. T1 (post-operatively baseline), T2 (after 20 min), and T3 (between 11 pm and midnight)	Salivary cortisol levels	=
	Prevalence of EEG beta activity (AAI vs. Ctrl)	EEG activity	increase (>14 Hz)
	Group × Time. Pre (T1, post-operatively baseline) and Post (T2, after 20 min) intervention	Cerebral oxygenation- HbO2 (%)	=
		Heart rate	increase
		SBP, DBP	=
		Oxygen saturation (%)	=
Vagnoli et al., 2015 ^#^	AAI vs. Ctrl (Before-During-After intervention)	Serum cortisol levels	During: decrease
Tsai et al., 2010	Group × Time (Pre-During-Post intervention)	SBP, DBP	SBP: decrease DBP: increase
		Heart rate	=
Braun et al., 2009	Group × Time (Pre-Post intervention)	Blood pressure	=
		Pulse rate	=
		Respiratory Rate	increase
Kaminski et al., 2002	AAI vs. Ctrl (Pre and Post intervention)	Heart rate	Pre: increase; Post: increase
		Blood pressure	n.a.
		Salivary cortisol	=
Havener et al., 2001 ^#^	Group × Time	Peripheral skin temperature	=
Hansen et al., 1999 ^#^	Group × Time (Pre-Post intervention)	SBP, DBP, MBP	=
		Heart rate	=
		Peripheral skin temperature	=
Nagengast et al., 1997	Group × Time	SBP, MBP	SBP overtime = decrease MBP overtime = decrease
		DBP	=
		Heart rate	decrease
		Peripheral Skin Temperature	=

**TABLE 5B:** Effect of AAI on stress and pain (physiological outcomes) (uncontrolled studies).

Silva and Osório, 2018	Pre–Post Intervention	Heart rate	=
		Blood pressure	=
Wu et al., 2002	Pre–Post Intervention	Heart Rate	=
		Respiratory Rate	=
		Oxygen saturation	=

*^#^Randomized controlled.*

*AAI, Animal-Assisted Intervention; Ctrl, Control Intervention; SBP, Systolic blood pressure; DBP, Diastolic blood pressure; MBP, mean arterial blood pressure.*

*=, Not significant changes; n.a., not available.*

Other physiological variables associated with arousal, such as systolic, diastolic, and mean arterial blood pressures were considered in seven controlled and one uncontrolled study (Nagengast et al., 1997; Hansen et al., 1999; Kaminski et al., 2002; Braun et al., 2009; Tsai et al., 2010; Calcaterra et al., 2015; McCullough et al., 2018; Silva and Osório, 2018). Five studies found no relevant changes in blood pressure, while in Tsai et al. (2010) research systolic blood pressure (SBP) decreased over time; DBP decreased during the intervention assisted by the dog, while this parameter increased after the control intervention suggesting that the mental activity associated with puzzle-solving may increase arousal, while AAI may help reducing it. The decrease in SBP after AAI lasted even few minutes after the intervention was over. In agreement with these observations, Nagengast et al. (1997) found a statistically significant decrease in SBP and MBP over time in patients involved in the AAI when compared to the control group, indicating that physiological arousal was moderated when the dog was present. One controlled study (Kaminski et al., 2002) collected, but did not show, the blood pressure data.

Four controlled studies examined cortisol levels (Kaminski et al., 2002; Calcaterra et al., 2015; Vagnoli et al., 2015; Branson et al., 2017) a hormone which is released under stressful conditions (Staufenbiel et al., 2013). The majority of those studies that have analyzed salivary cortisol found no relevant changes, while Vagnoli et al. (2015) found a relevant decrease in plasma cortisol levels in the group involved in activities with the dog.

Three controlled studies (Nagengast et al., 1997; Hansen et al., 1999; Havener et al., 2001) recorded peripheral skin temperature – a parameter used as an index of arousal – but found no significant changes. The respiratory rate was examined in one controlled and one uncontrolled study (Wu et al., 2002; Braun et al., 2009). While Wu et al. (2002) found no significant changes in respiratory rates, Braun et al. (2009) detected an increase in the respiratory activity (2 breaths/min) which may be considered as an indication of a state of excitement in the child or anticipation of seeing the dog in the hospital setting. One controlled and one uncontrolled study (Wu et al., 2002; Calcaterra et al., 2015) considered the oxygen saturation (%), finding no relevant changes. Furthermore, Calcaterra et al. (2015) recorded the cerebral oxygenation (HbO2%), finding no statistically relevant changes.

Finally, one controlled study recorded EEG activity (Calcaterra et al., 2015). The authors obtained a complete EEG recording reporting faster EEG diffuse beta activity (>14 Hz) in all children of the AAI group (0% vs. 100%, *p* < 0.001) after the entrance of the dog in the setting. Beta waves are high frequency, low-amplitude brain waves that are involved in conscious thought and logical thinking, and tend to have a stimulating effect. Low beta waves (12–15 Hz) are associated mostly with quiet, focused, introverted concentration (Saunders, 2007). In the AAI group, the presence of beta activity was correlated with an increase in attention, which could explain the finding of a higher threshold for pain sensitivity.

### Effect of Animal Assisted Interventions on Anxiety in Hospitalized Children and Adolescents

Eight of the 21 studies examined the effects of AAIs on children and adolescents’ anxiety levels. Hinic et al. (2019) found that, within the “pet” group, there was a significant difference in state anxiety scores before (*M* = 31; range = 20–46) and after (*M* = 25; range = 20–40; *p* < 0.001) the intervention. Post-intervention state anxiety scores were significantly lower in the AAI intervention group than in the comparison group (*p* = 0.002). McCullough et al. (2018) reported that children in both the intervention (*p* < 0.001) and control (*p* < 0.001) groups experienced significant reductions in their state anxiety throughout the study with medium effect sizes. Tsai et al. (2010), Barker et al. (2015), and Branson et al. (2017) found no evidence that AAI visits affected children’s anxiety. Anyway, in Barker and colleagues’ study the AAI group had significantly lower anxiety scores post-intervention (Table 6A). Both uncontrolled studies (Nammalwar and Rangeeth, 2018; Perez et al., 2019) found a statistically significant effect on anxiety levels after the AAI (Table 6B).

**TABLE 6A:** Effect of AAI on anxiety (controlled studies).

Study	Assessments/Analysis	Instruments/tool	Results
Hinic et al., 2019	Group × Time Pre and Post-intervention	STAI-C	↑
McCullough et al., 2018 ^#^	Group × Time. Pre–Post Intervention	STAI-C	↑
Branson et al., 2017 ^#^	Group × Time. Pre–Post Intervention	STAI-C	=
Barker et al., 2015 ^#^	AAI vs. Ctrl: Pre-Post Intervention	Anxiety rating scale*	=
Tsai et al., 2010	AAI vs. Ctrl: Post	STAI-C	=

**TABLE 6B:** Effect of AAI on anxiety (uncontrolled studies).

Perez et al., 2019	Pre-Post Intervention	VAS	↑
Nammalwar and Rangeeth, 2018	Pre-Post Intervention	RMS-PS	↑

*^#^Randomized controlled.*

*AAI, Animal-Assisted Intervention; Ctrl, Control Intervention; STAI-C, State-Trait Anxiety Inventory for Children (Spielberger, 1973); NRS-11, Numerical Rating Scale – 11-point, single item for anxiety (von Baeyer et al., 2009); VAS, Visual Analog Scale (Kain et al., 1997); RMS-PS, RMS Pictorial Scale (Shetty et al., 2015).*

*↑, Statistically significant improvement; =, not significant changes.*

### Other Outcomes

The effect of AAI on mood in hospitalized children and adolescents was explored in one controlled and one uncontrolled study (Kaminski et al., 2002; Silva and Osório, 2018). Results are mixed: while Silva and Osório (2018) found an improvement in one aspect of mood (i.e., irritation) following AAI, no changes were found in Kaminski et al.’s (2002) study, although, when child’s mood was reported by parents/caregivers, some positive results were found (i.e., happiness sub-item of a 5-point mood scale).

Preliminary evidence also shows the potential for AAI to improve wellbeing (Lindström Nilsson et al., 2020), communication and social interaction skills (Ávila-Álvarez et al., 2020), as well as experience of hospital stay (Lindström Nilsson et al., 2020), in children and adolescents, although further studies are needed to confirm these effects. Only two studies (McCullough et al., 2018; Silva and Osório, 2018) explored the effects of AAI on quality of life, although no changes were observed. Similar results were observed on depressive symptoms (Silva and Osório, 2018).

### Effect of Animal Assisted Interventions on Children’s Parents/Caregivers

Another aim of the current systematic review was to explore the effects of AAI on stress and burden, quality of life, mood, and level of satisfaction with hospitalization in parents/caregivers. However, very few studies assessed the impact of AAI on parents/caregivers. At present, preliminary evidence is encouraging: improvements in parent’s mood (mental confusion, tension; Silva and Osório, 2018), stress (communication sub-item; McCullough et al., 2018), and anxiety (Vagnoli et al., 2015; Silva and Osório, 2018) were observed.

### Risk of Bias

**FIGURE 2:**
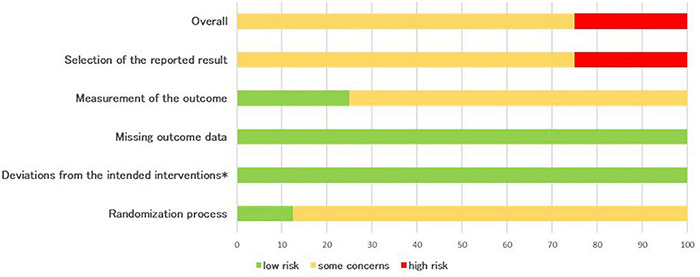
Percentages summary of risk-of-bias assessment using the RoB 2 tool (*effect of adhering to intervention).

An overall overview of the outcomes of the RoB2 assessment (randomized controlled studies) is presented in Figure 2. The majority of the studies selected raise some concerns (75% of studies) related to the different risks of bias (overall score). For the other studies (25%) high risk of bias were identified and this was mainly due to high-risk assessments in the selection of the reported result. Two domains displayed low risk-of-bias in all studies: missing outcome data and deviations from intended intervention, while some concerns are present in randomization process and measurement of the outcome domains (respectively, 87.5 and 75%).

**FIGURE 3:**
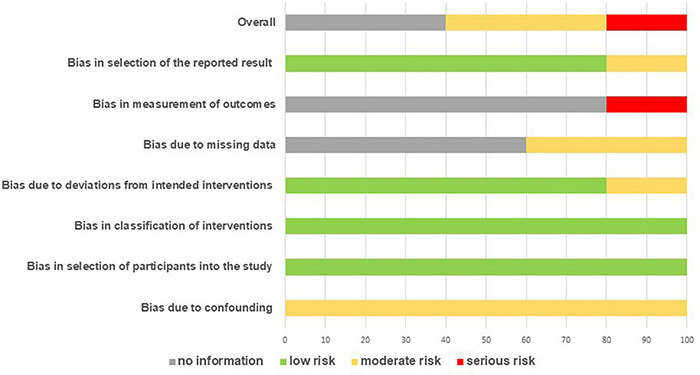
Percentages summary of risk-of-bias assessment using the ROBIN-I tool.

An overall overview of the outcomes of the ROBIN-I assessment is presented in Figure 3. As shown in Figure (overall score), moderate risk-of-bias was present in 40% of studies (bias due to confounding, 100%; deviations from intended interventions, 20%; missing data, 40% and selection of the reported result, 20%). Serious risk-of-bias (20%) was present in the assessed studies, mainly due to serious risk assessments in the measurement of outcomes. Not enough information was provided in 40% of the studies to assess the overall risk-of-bias, mainly due to a lack of information relative to missing data (60%) and measurement of outcomes (80%). Two domains displayed low risk-of-bias: selection of participants into the study and classification of intervention, while the low risk of bias due to deviations from intended interventions and selection of the reported result was present in 80% of the studies.

## Discussion

In this study, we have systematically reviewed both controlled and uncontrolled studies assessing the effectiveness of dog visits for improving emotional distress and the experience of hospitalization in children and adolescent patients in pediatric hospitals. Studies selected were rather dishomogeneous, both in terms of study design and type of activity offered. Notwithstanding such differences, the data overall indicate moderate effects of AAIs on stress/arousal and anxiety levels, as well as on pain perception in most of the pediatric sample. Preliminary evidence indicates positive effects also on mood, stress and anxiety in the caregivers, while no data concerning the hospital staff were reported.

Although we used very broad research criteria, such as searching for different species of pets and several outcomes for the three populations of interest (children, parents, and staff), we found exclusive dog use, while outcomes examined mainly focused on stress, pain and anxiety reduction. Our analysis reveals that other endpoints, such as patients’ mood and wellbeing, quality of life and caregivers’ burden, have been less frequently studied. Preliminary results on the latter measures are nonetheless promising: AAI showed a positive impact on reducing children’s irritation (Silva and Osório, 2018), improving wellbeing (Lindström Nilsson et al., 2020), enhancing communication and social interaction skills (Ávila-Álvarez et al., 2020) and ameliorating the experience of hospitalization (Lindström Nilsson et al., 2020). Concerning the impact of AAI on caregivers, we found some evidence of improvement in parents’ mood (Silva and Osório, 2018), stress (McCullough et al., 2018), and anxiety (Vagnoli et al., 2015; Silva and Osório, 2018).

One of the main outcomes which arouses interest is the effect of AAI on stress and pain in hospitalized pediatric patients. During our analysis, we first distinguished studies that included behavioral outcomes from those who considered physiological outcomes. In both cases, positive effects on stress and arousal were found. Almost all studies included confirmed a significant improvement in the levels of stress and pain after physical examinations and medical procedures using standardized tools (i.e., the Observational Scale of Behavioral Distress).

Physiological endpoints were in general more variable, and, as expected, were heavily influenced by individual thresholds and context-dependent factors. As an example, following AAI, one study (Calcaterra et al., 2015) found an increase in heart rate activity during post-operative awakening (commonly perceived as a stressful condition), and a similar excitatory response was also found in another study in which patients were in a quiet situation and exposed to no evident stressor (Kaminski et al., 2002). These two situations should be interpreted to indicate that the effects of an AAI depend on the specific context it is applied: in the first case, an increase in arousal was desirable to allow children to recover from surgery; in the second situation, the introduction of the dog caused an anticipatory excitement leading to activation. On the other hand, Nagengast et al. (1997) examined the heart rate of patients interacting with a dog during a medical examination, which can be considered as a stressful event (Coyne, 2006; Li et al., 2016), finding a significant decrease in heart activity and concluding that the intervention works as an inhibitor of the sympathetic nervous system. So, while the aim of the intervention may differ – promoting an adaptative response during surgery recovery, elicitating excitement, or reducing stress during a medical examination – measuring heart rate was effective in demonstrating the efficacy of AAIs in mediating and moderating stress levels in pediatric wards.

Those studies that examined salivary cortisol levels found no relevant pre-post intervention changes (Kaminski et al., 2002; Calcaterra et al., 2015; Branson et al., 2017). Vagnoli et al. (2015) tested cortisol in plasma and found a significant decrease in the AAI group, during blood testing, compared with the control group (Levine et al., 2007). It is worth reporting that, even if the use of plasma and salivary cortisol as an index of emotional distress is widespread (Buchanan et al., 1999; Melamed et al., 1999), methodological heterogeneity between the two may justify the lack of differences when using this endpoint. About this, Levine et al. (2007) reported that research measuring free cortisol using saliva requires some caution since, although saliva has advantages due to the ease of collection, the issues of compliance, variability, and identity between salivary and free cortisol can create drawbacks. Also, it is possible to hypothesize that in stressful situations, such as hospital settings, the baseline for cortisol may be elevated, making difficult to evaluate meaningful differences between groups. In another study with a sample of male children with insecure or avoidant attachment, Beetz et al. (2012) found that the effect of the presence of a dog during a stressful condition dropped significantly faster and to even lower cortisol levels in comparison with other conditions eliciting psychological support.

Other physiological variables associated with arousal, such as blood pressure, have yielded consistent results. Nagengast et al. (1997) found a statistically significant decrease in SBP and MBP over time in the AAI group (which can be interpreted as evidence of physiological arousal moderation during the dog’s presence), and Tsai et al. (2010) found blood pressure (SBP and DBP) decreasing after AAI but increasing after the control intervention, suggesting that the mental activity associated with puzzle-solving increases arousal, while AAI reduces it. In this regard, O’Haire et al. (2015) have demonstrated, using skin conductance analysis, that dogs’ presence can ameliorate the stressful nature of social interactions. These results are consistent with previous literature (Odendaal and Meintjes, 2003).

Furthermore, when Braun et al. (2009) examined the respiratory activity of children in acute pediatric care they found that the children’s respiratory activity increased with the excitement or anticipation of seeing the dog in the hospital setting. In their study, these authors concluded that pain reduction was four times greater in those children undergoing AAT as compared to those of the control group.

Interestingly, Calcaterra et al. (2015) have indicated that early post-operative intervention with AAT stimulation could facilitate rapid recovery of vigilance and activity after anesthesia with propofol, since the EEG analysis of children who benefited from the entrance of a dog in the examination room showed a faster EEG diffuse beta activity.

The effects of AAI on hospitalized children’s anxiety are likewise encouraging. The largest part of the studies we included showed a significant effect in reducing patients’ anxiety levels during hospitalization (Barker et al., 2015; McCullough et al., 2018; Nammalwar and Rangeeth, 2018; Hinic et al., 2019; Perez et al., 2019). Low levels of anxiety and cortisol levels may justify the lack of effects found in two of the selected studies (Tsai et al., 2010; Branson et al., 2017). As hypothesized by Friedmann (1995), companion animals can decrease anxiety and sympathetic nervous system arousal by providing a pleasant external focus for attention, promoting feelings of safety and providing a source of contact comfort. From a neurobiological point of view, this arousal-reducing effects may be mediated by changes in oxytocin levels that underly engaging human-dog relationships (Nagasawa et al., 2015). Attraction to dogs and positive/affectionate behaviors are likely to be elicited through their infantile physical and behavioral features (baby schema) emphasizing the central role of pets in human lives (Borgi and Cirulli, 2016).

Quality of the studies included (Risk of Bias analysis) reflect the fact that in the AAI field, which involves dynamic human-dog interactions, is often difficult to comply with strict research protocols (Borgi and Cirulli, 2015; Correale et al., 2017). An important future improvement might result from having the evaluator/scorer blind to the intervention, greatly reducing the risk of bias. Reviews like this one can provide important advise on dependent measure’s limits/opportunities, informing future research.

## Conclusion

Our revision confirms that AAIs are a suitable intervention in pediatrics’ wards. Compared to the previous review of Feng et al. (2021), here we analyzed different types of AAIs, including also activities, in addition to dog-assisted therapies. Data overall suggest that, when testing AAI efficacy, physiological indicators are more variable and less reliable than psychological and emotional measures (Khan and Farrag, 2000). Methodological issues may reduce the reliability of physiological indicators, and, although they are easy to be quantified, more research is needed to advance the field. As an example, although blood samples appear more reliable, the methodology for salivary cortisol measurement should be implemented in the future as this non-invasive sampling method should be preferred, especially within the pediatric population.

Behavioral measures have been more informative in assessing AAI effectiveness in reducing stress, pain, and anxiety. These instruments are amenable to self-administration, which makes them appealing, although they may suffer from respondent bias. Nonetheless, they are especially important as they contain important qualitative information. In an ideal setting, a good combination of those endpoints should be preferred. For this reasons, future programs in clinical practice should consider to incorporate physiological, behavioral and psychological measures of stress, anxiety and pain and include questionnaires for healthcare workers and caregivers to address acceptability and satisfaction towards the AAI’s intervention.

Data on the effectiveness of AAI on anxiety and stress are especially relevant within the framework of the coronavirus pandemic. Indeed, the risks and fears related to entry and stay in a hospital setting (separation from the parent, interruption of routine, invasive medical examinations, loss of sociality) are worsened by the fear of contagion, the climate of alarm, and the disruption in routine procedures (Crispo et al., 2020; Franchini et al., 2020; Garrafa et al., 2020; Wong et al., 2020; Candelaresi et al., 2021). To date, there is a pressing need for timely intervention to prevent these mental health morbidities and the role of pets in addressing mental health seems to be promising in this regard (Nagendrappa et al., 2020). There is a growing need to implement complementary therapies and interventions that may help pediatric patients feel more at ease in the hospital environment, making it as much as possible a suitable environment for children. AAI with dogs are appealing adjunct practices that may promote a more humanized health care, through arousal and anxiety reduction and distraction from painful procedures.

A limitation of this review concerns the limited number of children’s hospital populations surveyed in the articles collected. Here we evaluated the effects of AAIs in heterogeneous samples including both day hospital and chronically hospitalized children and did not distinguish between different medical conditions [i.e., Barker et al. (2015) performed the intervention including 31 different medical conditions]. Notwithstanding the sample limitations we still could report significant effects. Overall, we believe that AAI in the hospital setting is an important topic that deserves further attention in the future.

## Data Availability Statement

The original contributions presented in the study are included in the article/Supplementary Material, further inquiries can be directed to the corresponding author/s.

## Author Contributions

CC, FV, SC, FC, and SG conceived and designed the study. CC, BC, and MB extracted the data. CC, BC, MB, and CF wrote the first draft of the manuscript. FC and CC revised subsequent drafts, consolidated the manuscript, and contributed to its final version. All authors have approved the final article.

## Conflict of Interest

The authors declare that the research was conducted in the absence of any commercial or financial relationships that could be construed as a potential conflict of interest.

## Publisher’s Note

All claims expressed in this article are solely those of the authors and do not necessarily represent those of their affiliated organizations, or those of the publisher, the editors and the reviewers. Any product that may be evaluated in this article, or claim that may be made by its manufacturer, is not guaranteed or endorsed by the publisher.

## References

[B2] Abrahamson K. Cai Y. Richards E. Cline K. O’Haire M. E. (2016). Perceptions of a hospital-based animal assisted intervention program: an exploratory study. *Complement. Ther. Clin. Pract.* 25 150–154. 10.1016/j.ctcp.2016.10.003 27863605

[B3] Antonelli E. Vagnoli L. Ciucci E. Vernucci C. Lachi F. Messeri A. (2019). A comparison of nonpharmacologic interventions on the emotional state of children in the Emergency Department. *Pediatr. Emerg. Care* 35 81–88. 10.1097/PEC.0000000000000900 27749803

[B4] Ávila-Álvarez A. Alonso-Bidegain M. De-Rosende-Celeiro I. Vizcaíno-Cela M. Larrañeta-Alcalde L. Torres-Tobío G. (2020). Improving social participation of children with autism spectrum disorder: pilot testing of an early animal-assisted intervention in Spain. *Health Soc. Care Commun.* 28 1220–1229. 10.1111/hsc.12955 32022346

[B5] Barker S. B. Knisely J. S. Schubert C. M. Green J. D. Ameringer S. (2015). The effect of an animal-assisted intervention on anxiety and pain in hospitalized children. *Anthrozoos* 28 101–112. 10.1016/j.eujim.2016.05.005 32362955 PMC7185850

[B6] Beetz A. Julius H. Turner D. Kotrschal K. (2012). Effects of social support by a dog on stress modulation in male children with insecure attachment. *Front. Psychol.* 3:352. 10.3389/fpsyg.2012.00352 23162482 PMC3498889

[B7] Borgi M. Cirulli F. (2015). Attitudes toward animals among kindergarten children: species preferences. *Anthrozoos* 28, 45–59. 10.2752/089279315X14129350721939

[B8] Borgi M. Cirulli F. (2016). Pet face: mechanisms underlying human-animal relationships. *Front. Psychol.* 7:298. 10.3389/fpsyg.2016.00298 27014120 PMC4782005

[B9] Branson S. M. Boss L. Padhye N. S. Trötscher T. Ward A. (2017). Effects of animal-assisted activities on biobehavioral stress responses in hospitalized children: a randomized controlled study. *J. Pediatr. Nurs.* 36 84–91. 10.1016/j.pedn.2017.05.006 28888516

[B10] Braun C. Stangler T. Narveson J. Pettingell S. (2009). Animal-assisted therapy as a pain relief intervention for children. *Complement. Ther. Clin. Pract.* 15 105–109. 10.1016/j.ctcp.2009.02.008 19341990

[B11] Buchanan T. W. Al’Absi M. Lovallo W. R. (1999). Cortisol fluctuates with increases and decreases in negative affect. *Psychoneuroendocrinology* 24 227–241. 10.1016/s0306-4530(98)00078-x 10101730

[B12] Calcaterra V. Veggiotti P. Palestrini C. De Giorgis V. Raschetti R. Tumminelli M. (2015). Post-operative benefits of animal-assisted therapy in pediatric surgery: a randomised study. *PLoS One* 10:e0125813. 10.1371/journal.pone.0125813 26039494 PMC4454536

[B13] Candelaresi P. Manzo V. Servillo G. Muto M. Barone P. Napoletano R. (2021). The impact of Covid-19 lockdown on stroke admissions and treatments in Campania. *J. Stroke Cerebrovasc. Dis.* 30:105448. 10.1016/j.jstrokecerebrovasdis.2020.105448 33166767 PMC7640890

[B14] Chubak J. Hawkes R. Dudzik C. Foose-Foster J. M. Eaton L. Johnson R. H. (2017). Pilot study of therapy dog visits for inpatient youth with cancer. *J. Pediatr. Oncol. Nurs.* 34 331–341. 10.1177/1043454217712983 28614971 PMC6711573

[B15] Chur-Hansen A. McArthur M. Winefield H. Hanieh E. Hazel S. (2014). Animal-assisted interventions in children’s hospitals: a critical review of the literature. *Anthrozoos* 27 5–18. 10.2752/175303714x13837396326251

[B16] Cirulli F. Borgi M. Berry A. Francia N. Alleva E. (2011). Animal-assisted interventions as innovative tools for mental health. *Ann. Ist. Super. Sanità.* 47 341–348. 10.4415/ANN_11_04_04 22194067

[B17] Connor K. Miller J. (2000). Animal-assisted therapy: an in-depth look. *Dimens. Crit. Care Nurs.* 19 20–26. 10.1097/00003465-200019030-00006 11998003

[B18] Correale C. Crescimbene L. Borgi M. Cirulli F. (2017). Development of a Dog-Assisted Activity program in an elementary classroom. *Vet. Sci.* 4:62. 10.3390/vetsci4040062 29186915 PMC5753642

[B19] Coyne I. (2006). Children’s experiences of hospitalization. *J. Child Health Care* 10 326–336. 10.1177/1367493506067884 17101624

[B20] Crispo A. Montagnese C. Perri F. Grimaldi M. Bimonte S. Augustin L. S. (2020). COVID-19 emergency and post-emergency in Italian cancer patients: how can patients be assisted? *Front. Oncol.* 10:1571. 10.3389/fonc.2020.01571 32850461 PMC7431560

[B21] Davis T. N. Scalzo R. Butler E. Stauffer M. Farah Y. N. Perez S. (2015). Animal assisted interventions for children with autism spectrum disorder: a systematic review. *Educ. Train. Autism Dev. Disabil.* 43 316–329.

[B22] Feng Y. Lin Y. Zhang N. Jiang X. Zhang L. (2021). Effects of animal-assisted therapy on hospitalized children and teenagers: a systematic review and meta-analysis. *J. Pediatr. Nurs.* 60 11–23. 10.1016/j.pedn.2021.01.020 33582447

[B23] Fernandes S. C. Arriaga P. (2010). The effects of clown intervention on worries and emotional responses in children undergoing surgery. *J. Health Psychol.* 15 405–415. 10.1177/1359105309350231 20348361

[B24] Foster R. L. Varni J. W. (2002). Measuring the quality of children’s postoperative pain management: initial validation of the child/parent Total Quality Pain Management (TQPM™) instruments. *J. Pain Symp. Manag.* 23 201–210. 10.1016/s0885-3924(01)00411-0 11888718

[B25] Franchini S. Spessot M. Landoni G. Piani C. Cappelletti C. Mariani F. (2020). Stranger months: how SARS-CoV-2, fear of contagion, and lockdown measures impacted attendance and clinical activity during February and March 2020 at an urban Emergency Department in Milan. *Disaster Med. Public Health Prep.* 15 e33–e42. 10.1017/dmp.2020.265 32713377 PMC7588723

[B26] Francischinelli A. G. B. Almeida F. D. A. Fernandes D. M. S. O. (2012). Routine use of therapeutic play in the care of hospitalized children: nurses’ perceptions. *Acta Paul Enferm.* 25 18–23.

[B27] Friedmann E. (1995). “The role of pets in enhancing human well being: physiological effects,” in *The Waltham Book of Human–Animal Interaction: Benefits and Responsibilities of Pet Ownership*, ed. Robinson I. (Oxford: Pergamon Press), 33–53. 10.1016/b978-0-08-042284-8.50010-4

[B28] Garrafa E. Levaggi R. Miniaci R. Paolillo C. (2020). When fear backfires: emergency department accesses during the Covid-19 pandemic. *Health Policy* 124 1333–1339. 10.1016/j.healthpol.2020.10.006 33148454 PMC7584647

[B29] Gilmer M. J. Baudino M. N. Goddard A. T. Vickers D. C. Akard T. F. (2016). Animal-assisted therapy in pediatric palliative care. *Nurs. Clin.* 51 381–395. 10.1016/j.cnur.2016.05.007 27497015

[B30] Hansen K. M. Messinger C. J. Baun M. M. Megel M. (1999). Companion animals alleviating distress in children. *Anthrozoos* 12 142–148. 10.2752/089279399787000264

[B31] Havener L. Gentes L. Thaler B. Megel M. Baun M. Driscoll F. (2001). The effects of a companion animal on distress in children undergoing dental procedures. *Issues Compr. Pediatr. Nurs.* 24 137–152. 10.1080/01460860118472 11817428

[B32] Hinic K. Kowalski M. O. Holtzman K. Mobus K. (2019). The effect of a pet therapy and comparison intervention on anxiety in hospitalized children. *J. Pediatr. Nurs.* 46 55–61. 10.1016/j.pedn.2019.03.003 30852256

[B33] Italian National Guidelines for Animal Assisted Interventions (2015). *Agreement Between the Italian Government, the Regions and the Autonomous Provinces of Trento and Bolzano.* Available online at: http://www.salute.gov.it/imgs/C_17_opuscoliPoster_276_allegato.pdf (accessed March 25, 2015).

[B34] Jay S. M. Elliott C. (1986). *Observation Scale of Behavioral Distress-Revised: Information, Procedures, Definitions of Behaviors, OSBD Interval Coding Form.* Los Angeles, CA: Children’s Hospital.

[B35] Jiang W. Kuchibhatla M. Cuffe M. S. Christopher E. J. Alexander J. D. Clary G. L. (2004). Prognostic value of anxiety and depression in patients with chronic heart failure. *Circulation* 110 3452–3456. 10.1161/01.CIR.0000148138.25157.F9 15557372

[B36] Kain Z. N. Mayes L. C. Cicchetti D. V. Bagnall A. L. Finley J. D. Hofstadter M. B. (1997). The Yale preoperative anxiety scale: how does it compare with a “gold standard”? *Anesth. Analg.* 85 783–788. 10.1097/00000539-199710000-00012 9322455

[B37] Kaminski M. Pellino T. Wish J. (2002). Play and pets: the physical and emotional impact of child-life and pet therapy on hospitalized children. *Child Health Care* 31 321–335. 10.1207/s15326888chc3104_5 33486653

[B38] Khan M. A. Farrag N. (2000). Animal-assisted activity and infection control implications in a healthcare setting. *J. Hosp. Infect.* 46 4–11. 10.1053/jhin.2000.0785 11023717

[B39] Kruger K. A. Serpell J. A. (2010). “Animal-assisted interventions in mental health: definitions and theoretical foundations,” in *Handbook on Animal-Assisted Therapy: Theoretical Foundations and Guidelines for Practice*, 3rd Edn, ed. Fine A. H. (San Diego, CA: Elsevier Inc.). 10.1177/1039856217749056

[B40] Levine A. Zagoory-Sharon O. Feldman R. Lewis J. G. Weller A. (2007). Measuring cortisol in human psychobiological studies. *Physiol. Behav.* 90 43–53. 10.1016/j.physbeh.2006.08.025 17055006

[B41] Li W. H. Chung J. O. K. Ho K. Y. Kwok B. M. C. (2016). Play interventions to reduce anxiety and negative emotions in hospitalized children. *BMC Pediatr.* 16:36. 10.1186/s12887-016-0570-5 26969158 PMC4787017

[B42] Liberati A. Altman D. G. Tetzlaff J. Mulrow C. Gotzsche P. C. Ioannidis J. P. (2009). The PRISMA statement for reporting systematic reviews and meta-analyses of studies that evaluate health care interventions: explanation and elaboration. *PLoS Med.* 6:e1000100. 10.1371/journal.pmed.1000100 PMC2707010 19621070

[B43] Lindström Nilsson M. Funkquist E. L. Edner A. Engvall G. (2020). Children report positive experiences of animal-assisted therapy in paediatric hospital care. *Acta Paediatr.* 109 1049–1056. 10.1111/apa.15047 31597211

[B44] Lipp M. E. N. Lucarelli M. D. M. (1998). *Escala de Stress Infantil (ESI).* São Paulo: Casa do Psicologo, 1998.

[B45] Lundqvist M. Carlsson P. Sjödahl R. Theodorsson E. Levin L. Å (2017). Patient benefit of dog-assisted interventions in health care: a systematic review. *BMC Complement. Med. Ther.* 17:358. 10.1186/s12906-017-1844-7 28693538 PMC5504801

[B46] McCullough A. Ruehrdanz A. Jenkins M. A. Gilmer M. J. Olson J. Pawar A. (2018). Measuring the effects of an animal-assisted intervention for pediatric oncology patients and their parents: a multisite randomized controlled trial. *J. Pediatr. Oncol. Nurs.* 35 159–177. 10.1177/1043454217748586 29268667

[B47] Melamed S. Ugarten U. Shirom A. Kahana L. Lerman Y. Froom P. (1999). Chronic burnout, somatic arousal and elevated salivary cortisol levels. *J. Psychosom. Res.* 46 591–598. 10.1016/s0022-3999(99)00007-0 10454175

[B48] Moher D. Liberati A. Tetzlaff J. Altman D. G. PRISMA Group (2009). Preferred reporting items for systematic reviews and meta-analyses: the PRISMA statement. *BMJ* 339:b2535. 10.1136/bmj.b2535 19622551 PMC2714657

[B49] Moher D. Shamseer L. Clarke M. Ghersi D. Liberati A. Petticrew M. (2015). Preferred Reporting Items for Systematic Review and Meta-Analysis Protocols (PRISMA-P) 2015 statement. *Syst. Rev.* 4:1. 10.1186/2046-4053-4-1 25554246 PMC4320440

[B50] Nagasawa M. Mitsui S. En S. Ohtani N. Ohta M. Sakuma Y. (2015). Oxytocin-gaze positive loop and the coevolution of human-dog bonds. *Science* 348 333–336. 25883356 10.1126/science.1261022

[B51] Nagendrappa S. Shoib S. Rehman S. Grigo O. Ransing R. (2020). Recognizing the role of animal-assisted therapies in addressing mental health needs during the COVID-19 pandemic. *Asian J. Psychiatr.* 53:102390. 10.1016/j.ajp.2020.102390 32882672 PMC7451050

[B52] Nagengast S. L. Baun M. M. Megel M. Leibowitz J. M. (1997). The effects of the presence of a companion animal on physiological arousal and behavioral distress in children during a physical examination. *J. Pediatr. Nurs.* 12 323–330. 10.1016/s0882-5963(97)80058-9 9420370

[B53] Nammalwar R. B. Rangeeth P. (2018). A bite out of anxiety: evaluation of animal-assisted activity on anxiety in children attending a pediatric dental outpatient unit. *J. Indian Soc. Pedod. Prev. Dent.* 36:181. 10.4103/JISPPD.JISPPD_54_18 29970636

[B54] Odendaal J. S. Meintjes R. A. (2003). Neurophysiological correlates of affiliative behaviour between humans and dogs. *Vet. J.* 165 296–301. 10.1016/s1090-0233(02)00237-x 12672376

[B55] O’Haire M. E. McKenzie S. J. Beck A. M. Slaughter V. (2015). Animals may act as social buffers: skin conductance arousal in children with autism spectrum disorder in a social context. *Dev. Psychobiol.* 57 584–595. 10.1002/dev.21310 25913902 PMC8447904

[B56] Perez M. Cuscaden C. Somers J. F. Simms N. Shaheed S. Kehoe L. A. (2019). Easing anxiety in preparation for pediatric magnetic resonance imaging: a pilot study using animal-assisted therapy. *Pediatr. Radiol.* 49 1000–1009. 10.1007/s00247-019-04407-3 31030334

[B57] Ravindran A. V. da Silva T. L. (2013). Complementary and alternative therapies as add-on to pharmacotherapy for mood and anxiety disorders: a systematic review. *J. Affect Disord.* 150 707–719. 10.1016/j.jad.2013.05.042 23769610

[B58] Saunders W. B. (2007). “Chapter 10 – Epilepsy,” in *Clinical Neurology for Psychiatrists (Sixth Edition)*, ed. Kaufman D. M. (Amsterdam: Elsevier), 203–240.

[B59] Shamseer L. Moher D. Clarke M. Ghersi D. Liberati A. Petticrew M. (2015). Preferred Reporting Items for Systematic Review and Meta-Analysis Protocols (PRISMA-P) 2015: elaboration and explanation. *BMJ* 349:g7647. 10.1136/bmj.g7647 25555855

[B60] Sherman S. A. Eisen S. Burwinkle T. M. Varni J. W. (2006). The PedsQL™ present functioning visual analogue scales: preliminary reliability and validity. *Health Qual. Life Outcomes* 4:75. 10.1186/1477-7525-4-75 17020606 PMC1617086

[B61] Shetty R. M. Khandelwal M. Rath S. (2015). RMS Pictorial Scale (RMS-PS): an innovative scale for the assessment of child’s dental anxiety. *J. Indian Soc. Pedod. Prev. Dent.* 33 48–52. 10.4103/0970-4388.149006 25572374

[B62] Silva N. B. Osório F. L. (2018). Impact of an animal-assisted therapy programme on physiological and psychosocial variables of paediatric oncology patients. *PLoS One* 13:e0194731. 10.1371/journal.pone.0194731 29617398 PMC5884536

[B63] Sobo E. J. Eng B. Kassity-Krich N. (2006). Canine visitation (pet) therapy: pilot data on decreases in child pain perception. *J. Holist Nurs.* 24 51–57. 10.1177/0898010105280112 16449747

[B64] Spielberger C. D. (1973). *Preliminary Test Manual for the State-Trait Anxiety Inventory for Children.* Palo Alto, CA: Consulting Psychologists Press. Inc.

[B65] Staufenbiel S. M. Penninx B. W. Spijker A. T. Elzinga B. M. van Rossum E. F. (2013). Hair cortisol, stress exposure, and mental health in humans: a systematic review. *Psychoneuroendocrinology* 38 1220–1235. 10.1016/j.psyneuen.2012.11.015 23253896

[B66] Sterne J. A. Hernán M. A. Reeves B. C. Savović J. Berkman N. D. Viswanathan M. (2016). ROBINS-I: a tool for assessing risk of bias in non-randomised studies of interventions. *BMJ* 12:i4919. 10.1136/bmj.i4919 27733354 PMC5062054

[B67] Sterne J. A. C. Savović J. Page M. J. Elbers R. G. Blencowe N. S. Boutron I. (2019). RoB 2: a revised tool for assessing risk of bias in randomised trials. *BMJ* 366:l4898. 10.1136/bmj.l4898 31462531

[B68] Taniguchi S. Martins R. M. Vogel C. Ropero J. Mason R. (2015). Initial palliative care drugs’ side effect. *Eur. Psychiatry.* 30 (Suppl. 1) 1507. 10.1016/s0924-9338(15)32058-7

[B69] Tripodi M. Siano M. A. Mandato C. De Anseris A. G. E. Quitadamo P. Nuzio S. G. (2019). Humanization interventions in general pediatric wards: a systematic review. *Eur. J. Pediatr.* 178 607–622. 10.1007/s00431-019-03370-3 30949888

[B70] Tsai C. C. Friedmann E. Thomas S. A. (2010). The effect of animal-assisted therapy on stress responses in hospitalized children. *Anthrozoos* 23 245–258. 10.2752/175303710x12750451258977

[B71] Uglow L. S. (2019). The benefits of an animal-assisted intervention service to patients and staff at a children’s hospital. *Br. J. Commun. Nurs.* 28 509–515. 10.12968/bjon.2019.28.8.509 31002549

[B72] Urbanski B. L. Lazenby M. (2012). Distress among hospitalized pediatric cancer patients modified by pet-therapy intervention to improve quality of life. *J. Pediatr. Oncol. Nurs.* 29 272–282. 10.1177/1043454212455697 22907682

[B73] Vagnoli L. Caprilli S. Vernucci C. Zagni S. Mugnai F. Messeri A. (2015). Can presence of a dog reduce pain and distress in children during venipuncture? *Pain Manag. Nurs.* 16 89–95. 10.1016/j.pmn.2014.04.004 25439114

[B74] von Baeyer C. L. Spagrud L. J. McCormick J. C. Choo E. Neville K. Connelly M. A. (2009). Three new datasets supporting use of the Numerical Rating Scale (NRS-11) for children’s self-reports of pain intensity. *Pain* 143 223–227. 10.1016/j.pain.2009.03.002 19359097

[B75] Waite T. C. Hamilton L. O’Brien W. (2018). A meta-analysis of Animal Assisted Interventions targeting pain, anxiety and distress in medical settings. *Complement. Ther. Clin. Pract.* 33 49–55. 10.1016/j.ctcp.2018.07.006 30396626

[B76] William Li H. C. Lopez V. Lee T. L. I. (2007). Effects of preoperative therapeutic play on outcomes of school-age children undergoing day surgery. *Res. Nurs. Health* 30 320–332. 10.1002/nur.20191 17514706

[B77] Wong D. L. Baker C. M. (1988). Pain in children: comparison of assessment scales. *Pediatr. Nurs.* 14 9–17. 3344163

[B78] Wong L. E. Hawkins J. E. Murrell K. L. (2020). Where are all the patients? Addressing Covid-19 fear to encourage sick patients to seek emergency care. *NEJM Catal. Innov. Care Deliv.* [Epub ahead of print].

[B79] Wu A. S. Niedra R. Pendergast L. McCrindle B. W. (2002). Acceptability and impact of pet visitation on a pediatric cardiology inpatient unit. *J. Pediatr. Nurs.* 17 354–362. 10.1053/jpdn.2002.127173 12395303

