# Supplementary material for: Improving the Emotional Distress and the Experience of Hospitalization in Children and Adolescent Patients Through Animal Assisted Interventions: A Systematic Review
Source: Front Psychol. 2022 Mar 4;13:840107. doi: 10.3389/fpsyg.2022.840107 (PMC8934415; doi:10.3389/fpsyg.2022.840107)
Supplement: Supplementary file 1 [file Data_Sheet_1.docx]

**Scopus**

((TITLE-ABS-KEY({human-pet bond*} OR {human-animal bond*} OR {human-dog bond*} OR {human-pet interaction} OR {human-animal interaction} OR {human-dog interaction} OR {human-pet relation*} OR {human-animal relation*} OR {human-dog relation*} OR pet-assisted)) OR (TITLE-ABS-KEY(pet-facilitated OR animal-assisted OR animal-facilitated OR dog-assisted OR dog-facilitated OR {pet therapy} OR {therapy animal} OR {therapy dog} OR {animal visit*} OR {pet visit*} OR {dog visit*} OR {canine visit*}))) AND ((TITLE-ABS-KEY(hospital OR clinic OR outpatient OR inpatient OR hospice OR {health care} OR {care setting} OR {intensive care} OR {acute care} OR {palliative care} OR nursing OR surgery OR oncology OR cardiology OR neurorehabilitation OR neurology OR {medical procedure} )) OR (TITLE-ABS-KEY(radiology OR {blood sampl*} OR {blood test} OR {blood collection} OR venipuncture OR dental OR oral OR exam OR examination OR assessment OR test))) AND (TITLE-ABS-KEY(child* OR adolescent OR pediatric))

*TITLE-ABS-KEY: Title, Abstract and Keywords*
